# V-pattern with diplopia following bilateral lateral rectus recession in intermittent exotropia

**DOI:** 10.3389/fopht.2026.1982180

**Published:** 2026-10-26

**Authors:** Jaime Tejedor, Francisco J. Gutiérrez-Carmona

**Affiliations:** 1 Department of Ophthalmology, Hospital Ramón y Cajal, Madrid, Spain; 2 Department of Neuroscience, Universidad Autónoma de Madrid, Madrid, Spain; 3 Instituto de Investigación Oftalmológica Ramón Castroviejo, Madrid, Spain

**Keywords:** abnormal head position, diplopia, intermittent exotropia, strabismus, V pattern

## Abstract

**Purpose:**

To report a case series of patients presenting with diplopia, V-pattern with esotropia/esophoria, and chin-down head position after bilateral lateral rectus recession (BLR) for the treatment of intermittent exotropia (IXT).

**Methods:**

The historical data of patients were recorded, in addition to variables of routine ophthalmological evaluation, including age at onset of IXT; age at BLR; preoperative deviation; magnitude of recessions; postoperative deviation and stereoacuity; ductions and versions; onset and degree of alphabet pattern, diplopia, and chin-down/abnormal head position (AHP); time between the initial BLR and second surgery (inferior displacement of the medial rectus muscles); and degree of correction of the V-pattern, AHP, and diplopia.

**Results:**

Six patients were identified. After BLR, two patients had small-angle esotropia, and four had esophoria. The time between surgery and V-pattern development/chin-down position ranged from 2 months to 2 years. Diplopia occurred in five patients. We did not observe inferior oblique overaction, but small subjective excyclotorsion (3°–5°) was detected in four of the six patients. The second surgery occurred around the age of 7 years, except in one patient aged 28 years. The V-pattern and abnormal head position were corrected after medial rectus infraplacement. Orthophoria was observed in five patients and esophoria of 4 prism diopters (PD) in one patient. Subjective torsion was neutralized in only one patient.

**Conclusion:**

V-pattern and chin-down position with diplopia may occur after BLR for IXT. We describe successful surgical correction of diplopia and V-pattern/abnormal head position by infraplacement of medial rectus muscles.

## Introduction

Surgery for intermittent exotropia (IXT) consists of bilateral lateral rectus recession (BLR) or unilateral lateral rectus recession-medial rectus resection (R&R). In the basic type, the required amount of BLR might need to be increased to achieve results comparable to those obtained with R&R surgery (1). In patients with distance–near disparity and a true high accommodative convergence/accommodation (AC/A) ratio, standard surgery to correct distance exodeviation may lead to near esotropia (1–3).

We have treated six patients, over the last 8 years, who underwent BLR surgery for IXT correction and subsequently developed a V-pattern esotropia/esophoria, chin-down abnormal head position, and intermittent diplopia, which may be attributed to various mechanisms (4–7). We examined the characteristics of these patients, influential variables, and the outcome of surgery performed to treat this pattern.

## Methods

The study and data collection were conducted under Institutional Review Board approval (‘Comité Ético de Investigación con Medicamentos’), and informed consent was obtained from the participants or their parents/caregivers. We performed a retrospective collection of data in patients who underwent surgery to correct a V-pattern developed after BLR for the treatment of intermittent exotropia.

A routine, complete ophthalmologic evaluation was carried out for each patient, including assessment of visual acuity, refraction, simultaneous and alternate prism and cover tests, AC/A ratio (gradient method with a +3.00 D lens), ductions, versions, subjective cyclotorsion, stereoacuity, and biomicroscopy. A 9-gaze motility examination was performed preoperatively before BLR and also before and after the second corrective surgery.

Variables recorded included age at onset of IXT; age at the initial surgery (BLR); preoperative deviation frequency and magnitude; ductions and versions; the magnitude of recessions; postoperative deviation and stereoacuity at 2 months; time and age when the V-pattern started after BLR; measurement of the V-pattern; onset of diplopia; the presence of chin-down/abnormal head position (AHP); the interval between the initial and second surgery to treat the V-pattern/AHP by inferior transposition of medial rectus muscles; and the degree of correction of the V-pattern, AHP, and diplopia after the second surgery. In all cases, the second surgery consisted of a half-tendon inferior transposition of both medial rectus muscles, except in case 2, where a full-tendon-width displacement was performed.

## Results

**Table 1** presents the characteristic variables. Three of the six patients underwent surgery for intermittent exotropia (IXT) at our hospital, and the other three were treated at other centers. Bilateral lateral rectus recession (BLR) was performed on children aged 6 to 8 years. Preoperative deviations ranged from −28 to −35 prism diopters (PD), without an alphabetic pattern. The distance–near disparity disappeared after eye patching, while the near deviation did not increase with +3.00 lenses (no high AC/A ratio), and control of deviation was fair to poor preoperatively. The amount of recession was 8 to 8.5 mm for each lateral rectus. In the three patients who underwent BLR for IXT at another center, we had access to the clinical records provided by the patients, which included preoperative examinations of ductions and versions, and we conducted the 9-gaze examinations ourselves before and after the second surgery.

**Table 1:** Characteristics of patients.

	Age at onset years months	Age at initial surgery years months	Deviation pre-BLR (distance)^*^ PD	Amount of recession (BLR) mm	Deviation post-BLR (distance) PD	SA post-BLR arc seconds	Age at onset of V/chin-down position years months	IO	Deviation- upgaze- primary position- downgaze PD Excyclo degrees	VA Snellen (decimal) OD/OS	Refraction Diopters Sphere Cylinder Axis	Age at second surgery years months	Deviation after second surgery- upgaze- primary position- downgaze PD Excyclo degrees	Chin-down correction
1	3 y 6 m	8 y	-28	8 HB	SPCT +8 APCT + 14	200	10 y	√	+5 +10 +24 Exc 5°	1.0/1.0	-3.75 -3.75	28 y	0 0 + 4 0+ Exc 4°	√
2	3 y 8 m	6 y 4 m	-30	8 DS	SPCT 0+ APCT +10	60	7 y 1 m	√	0 0 to +10 +25 to +30 Exc 5°	1.0/1.0	-0.75x5 -0.75x5	7 y 9 m	0- 0 0 + 4 dis Exc 2°	√
3	3 y 10 m	6 y 9 m	-30	8.5 DS	SPCT 0+ APCT +14	60	7 y 5 m	√	0 0 to +14 +20	1.0/1.0		7 y	0 0 0+	√
4	3 y	6 y 7 m	-35	8.5 HB	SPCT 0+ APCT +15		7 y 1 m	√	0- 0 to +10 +24 Exc 3°	1.0 dif/1.0	-0.5 -1x10 -0.5 -0.75x170	7 y 8 m	0 0+ 0+ No exc	√
5	4 y	7 y	-30	8 HB	SPCT +4 APCT + 8	100	7 y 9 m	√	0+ +4 +20 Exc 5°	1.0/1.0	-0.5 -0.5-1x15	8 y 6 m	0 0+ 0 dis+2 Exc 3°	√
6	2 y 10 m	5 y 11 m	-35	8.5 HB	SPCT 0 APCT 0+	60	7 y	√	0- 0+ +15	1.0/0.9	-0.75 -0.5 -1.75 x 165	7 y 10 m	0 0+ 0+	√

* Same as near deviation after eye patch.

y, years; m, months; PD, prism diopters; BLR, bilateral lateral rectus recession; DS, direct scleral fixation; HB, hang-back recession; SPCT, simultaneous prism and cover test; APCT, alternate prism and cover test; SA, stereoacuity; V, V pattern; IO, inferior oblique function; Exc, excyclotorsion; VA, visual acuity; OD, right eye; OS, left eye; dif, with difficulty; √ = normal examination; + sign means esotropia (ET) and – sign means exotropia (XT) in deviation measurements; dis, dissociating.

Following BLR, two patients developed esotropia, while four remained orthotropic with small esophoria in primary gaze (see **Figures 1**, **2**). The interval between surgery and the emergence of a V-pattern or chin-down posture varied from 2 months to 2 years. Diplopia was reported in five patients in primary position and downgaze (constant in two and intermittent in three patients). No evidence of inferior oblique overaction, oblique muscle dysfunction, hyperdeviation, or dissociated deviation was found. Four of the six patients exhibited subjective excyclotorsion of 3°–5° as measured by the double Maddox rod test, confirmed by fundus objective torsion. Visual acuity and stereoacuity were good in all but one patient, who had a manifest small-angle esotropia and gross stereoacuity. Mild-to-moderate myopia was corrected in every case. All patients exhibited a chin-down position with a V-pattern after BLR. For the correction of the V-pattern, we performed surgery at about age 7, except in one patient aged 28 years. After medial rectus infraplacement—which included a 1–2 mm recession in cases 1 and 5—the V-pattern and abnormal head position resolved, with orthophoria in primary gaze in five patients and orthotropia with esophoria of 4 PD in one patient. Excyclotorsion was fully neutralized in one patient.

**Figure 1:**
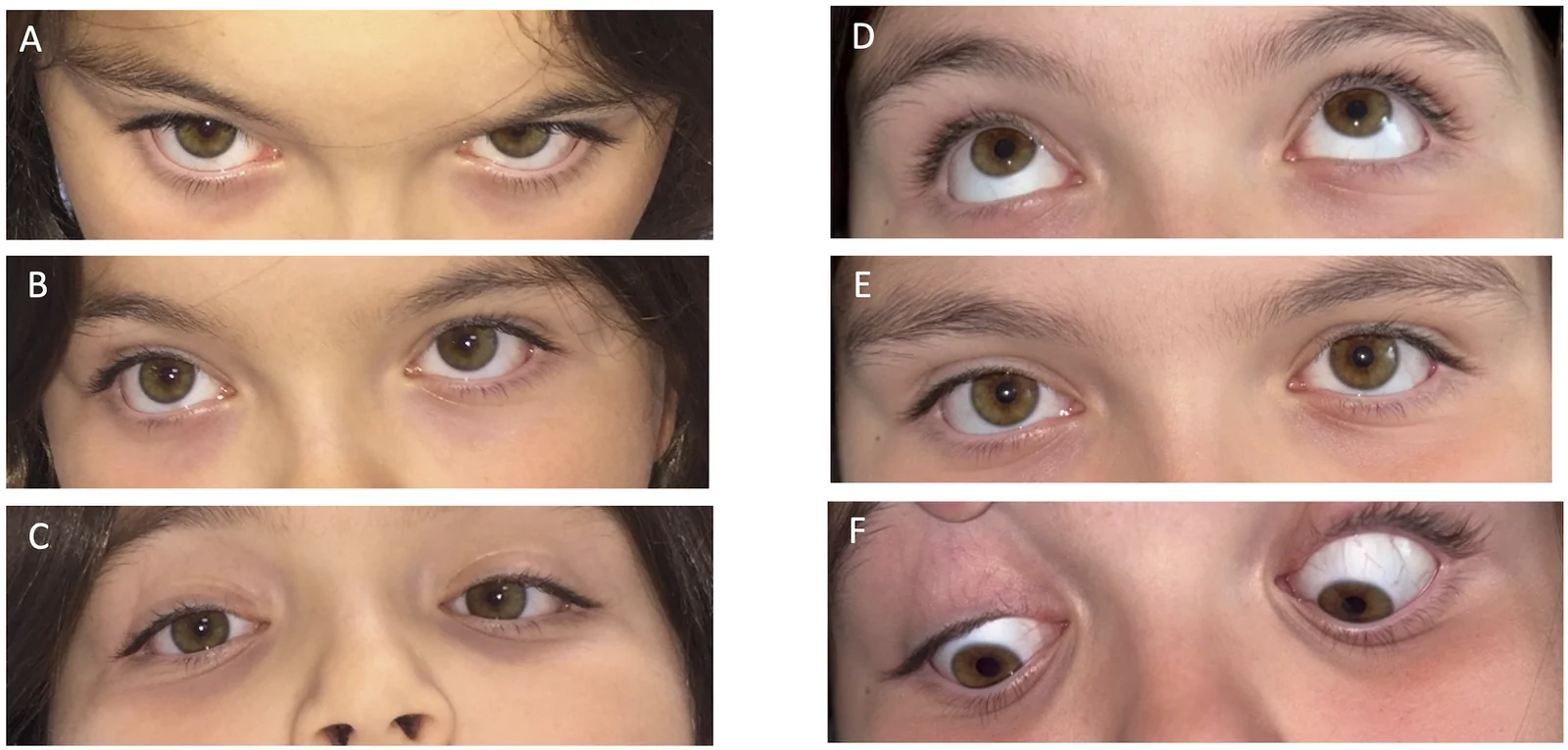
V-pattern and chin-down abnormal head position in an 8-year-old girl (up: -5 PD; down: +25 PD) after bilateral lateral rectus recession for the treatment of intermittent exotropia **(A–C)**, successfully corrected with inferior displacement of medial rectus muscles **(D–F)**.

**Figure 2:**
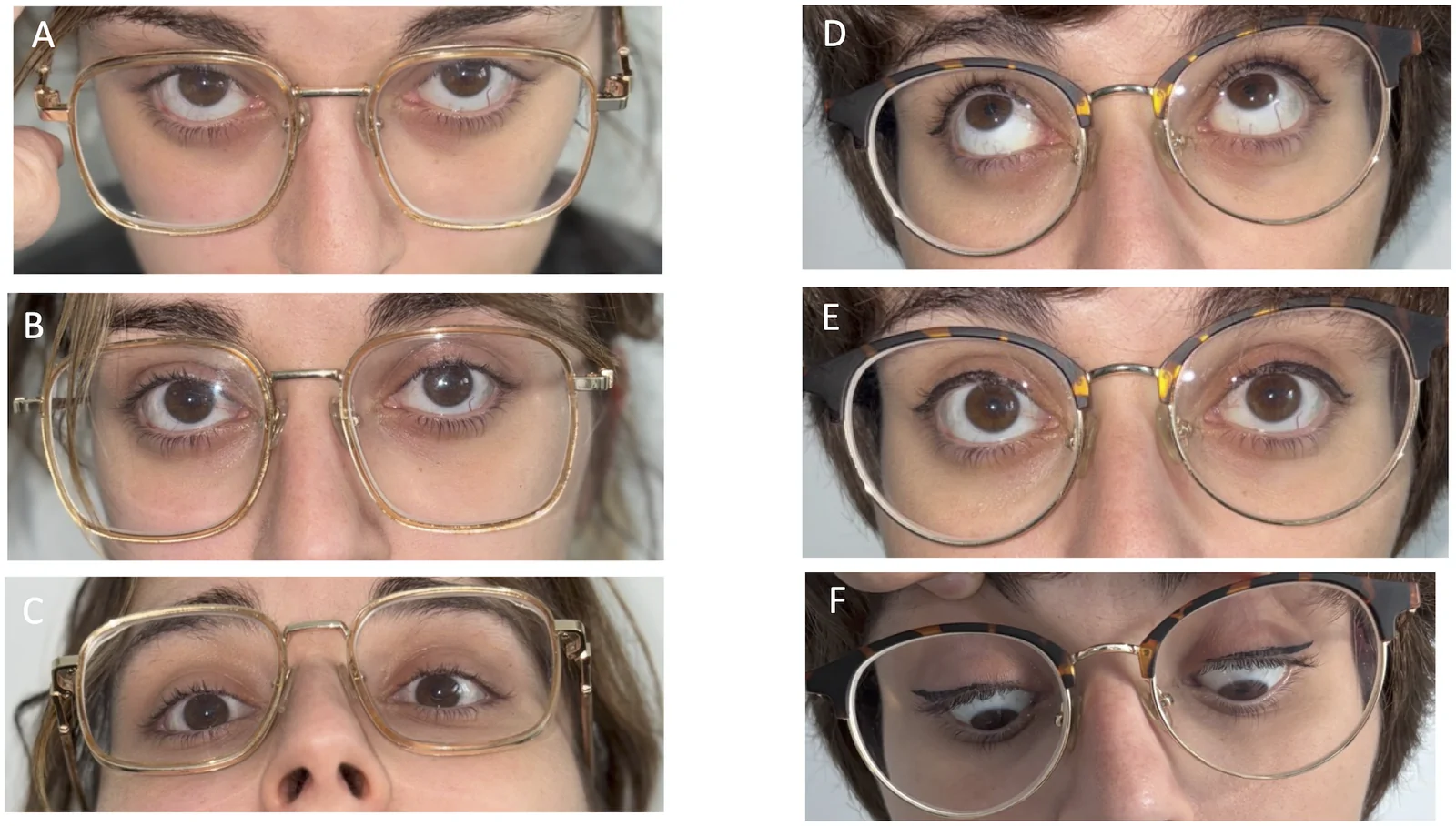
V-pattern and chin-down abnormal head position in a 28-year-old woman (up: -4 PD; down: +20 PD) after bilateral lateral rectus recession for the treatment of intermittent exotropia **(A–C)**, successfully corrected with inferior displacement of medial rectus muscles **(D–F)**.

## Discussion

We have described six cases of V-pattern esotropia/esophoria and abnormal chin-down position occurring 2 months to 2 years after BLR surgery for IXT that were treated successfully with bilateral inferior displacement of the medial rectus muscles.

A plausible explanation for this finding is that consecutive esotropia or esophoria occurs after BLR. Esotropia was found in two of the six patients and esophoria in the remaining four. None of the patients showed a high AC/A ratio before surgery, indicating a low risk of near esotropia after standard surgical correction.

Miller and Guyton confirmed that the loss of fusion predisposes the ocular motor system to the development of A or V patterns (4, 5). Esotropia alters or disrupts fusion, which could lead to ‘sensory’ torsional deviation (torsional equivalent to horizontal or vertical deviation that appears after the loss of fusion). Torsion rotates the planes of action of the vertical rectus muscles and causes apparent overaction of the inferior oblique muscles by rotation of the planes of action of the horizontal rectus muscles. In theory, excyclotorsion converts the medial recti into partial elevators and the lateral recti into partial depressors, resulting in elevation in adduction, resembling inferior oblique overaction (6). The superior rectus muscles would acquire a partial abductor action, and the inferior rectus muscles would acquire a partial adductor action, contributing to the V-pattern. In the clinical evaluation of our case series, we did not observe inferior oblique overaction, but small subjective excyclotorsion was detected in four cases. Kushner concluded that although ocular torsion may contribute to A or V-patterns, it is probably not the major cause (6).

Another explanation is that the lateral rectus (LR) muscles could have been infraplaced when BLR was performed, which might have caused a V-pattern. For hang-back recessions, we mark the center of the muscle scleral insertion before disinsertion, and the suture is attached to that point. Three patients underwent BLR at a different center, where a classical recession with direct scleral fixation was used, making inadvertent inferior displacement of the LR muscles less likely. However, this assumption remains speculative because we do not have direct evidence of the LR muscle positions after recessions. No associated vertical deviations were observed.

When LR muscles are recessed, the connection of their pulley to the inferior oblique/inferior rectus might cause the muscle to descend, especially relative to the medial rectus position, and induce a V-pattern. A- and V-patterns, when not preceded by surgery, may be caused by pulley heterotopia or abnormal position or direction of eye muscle paths. An LR pulley shifted inferiorly (e.g., in adduction and infraduction) has been described in strabismus with V-pattern incomitance (7). Furthermore, the LR muscles have been suggested to act more in elevation. After a recession, their action in depression may be decreased even more and cause or augment esotropia.

Elkamshoushy et al (8) described cases of pattern strabismus with consecutive esodeviation after bilateral lateral rectus muscle recession for intermittent exotropia. However, they reported a few cases with an A pattern whereas, in our case series, only patients with a V-pattern have been described. In their study, consecutive esotropia was always present, but we found esotropia after BLR in only two cases, with the remaining four cases only having esophoria. Finally, they treated the esodeviation surgically and we performed infraplacement of the medial rectus muscles to correct the V-pattern.

A potential criticism is that inferior transposition of the medial rectus muscles could induce or increase excyclotorsion. It has been reported mainly in the context of bilateral trochlear nerve palsy (9), but clinically relevant excyclotorsion is not associated with infraplacement of the medial rectus muscles carried out frequently in V-pattern cases by strabismus surgeons, probably because the amount of torsional change is small (no more than 2° according to our calculation). Thus, treating alphabet patterns with medial rectus muscle transposition usually does not cause adverse symptomatic torsional outcomes (10, 11). We did not observe either an increase or induction of excyclotorsion in the series of patients reported. It has been suggested that V-pattern (exotropia) with minimal or no inferior oblique overaction could be managed by bilateral inferior oblique weakening (myectomy) (12). In that study, a successful outcome was achieved in 72% of patients using inferior oblique myectomy compared to 56% of those treated by bilateral lateral rectus recession combined with full-tendon-width upward transposition. However, in the myectomy group 22% had overcorrection with a consecutive A pattern. These results are not directly comparable to the results in our cohort because our patients underwent inferior transposition of both medial rectus muscles rather than bilateral upward transposition of the lateral rectus.

The V-pattern may be overlooked in these children, particularly when the chin-down position is not remarkable. R&R surgery for IXT never preceded the V-pattern and chin-down position in this case series. A limitation of this manuscript is the small number of subjects included, in part due to the relatively low frequency of this finding.

In conclusion, a V-pattern with diplopia and chin-down position may be found after BLR surgery for intermittent exotropia and may respond successfully to surgical correction.

## Data Availability

The original contributions presented in the study are included in the article/supplementary material. Further inquiries can be directed to the corresponding author.
